# Les métastases ovariennes d'origine biliaire: 2 cas avec revue de la littérature

**DOI:** 10.11604/pamj.2013.16.44.787

**Published:** 2013-10-07

**Authors:** Imane Kamaoui, Mustapha Maaroufi, Benjalloun el Bachir, Hanane Ouzaa, Siham Tizniti

**Affiliations:** 1 Service de Radiologie, CHU Hassan II, Maroc; 2 Service de chirurgie viscérale, CHU Hassan II, Maroc

**Keywords:** Cancer de la vésicule biliaire, métastase ovarienne, masses ovariennes bilatérales, imagerie, Gallbladder cancer, ovarian metastasis, bilateral ovarian masses, imagery

## Abstract

Les ovaires constituent un site fréquent de métastases. L'origine gastrique prédomine. Les métastases ovariennes d'origine biliaire sont rarement rapportées dans la littérature. Les auteurs rapportent deux cas de métastases ovariennes d'origine vésiculaire chez des patientes âgées respectivement de 63 et 40 ans. Le diagnostic de ces métastases ovariennes était concomitant avec le cancer d'origine dans le premier cas, et a survenu à distance de l'atteinte initiale dans le deuxième cas. Le diagnostic est suggéré sur les données radiologiques et confirmé histologiquement. Les métastases ovariennes d'origine biliaire sont rarement rapportées dans la littérature. L'atteinte ovarienne pose un problème de diagnostic différentiel avec une atteinte ovarienne primitive surtout si l'atteinte ovarienne précède les manifestations biliaires. L'imagerie joue un rôle important et oriente sur le caractère secondaire de l'atteinte ovarienne.

## Introduction

Les ovaires constituent un site fréquent de métastases. Environ 5 à 15% des lésions ovariennes malignes sont des métastases [1]. La majorité des sites primitifs sont d'origine gastro-intestinale notamment l'estomac et le colon. L'origine biliaire est rarement décrite dans la littérature. La survenue de ces métastases à distance de la découverte de la tumeur d'origine pose un véritable problème de diagnostic différentiel avec une tumeur ovarienne primitive. Les auteurs rapportent 2 cas de métastases ovariennes d'origine biliaire.

## Patient et observation

### Cas 1

**Figure 1:**
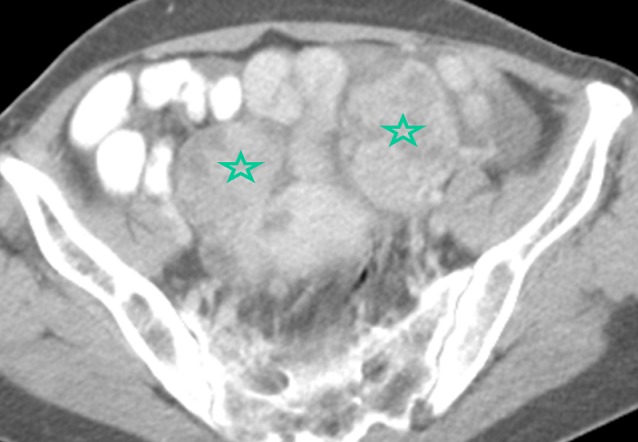
Image scannographique en coupe axiale qui montre deux masses pelviennes bilatérales suggérant des masses ovariennes (étoiles)

**Figure 2:**
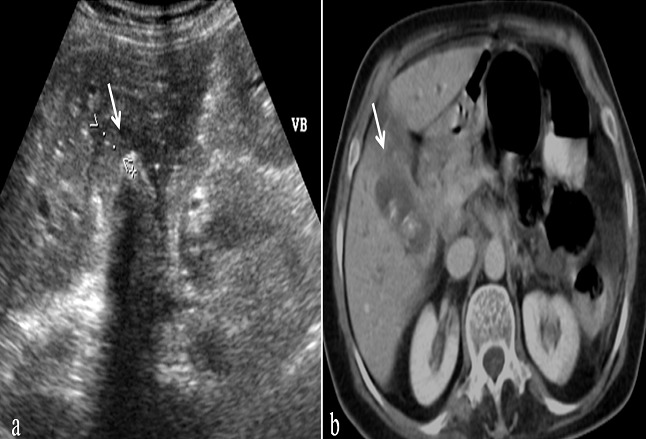
Coupe échographique (a) et scannographique (b) montrant un épaississement de la paroi vésiculaire en faveur d'une cholécystite chronique lithiasique (flèche).

Patiente âgée de 63 ans, qui présente depuis 2 mois des douleurs abdominales diffuses avec sensation de pesanteur pelvienne. L'examen clinique trouve une patiente en assez bon état général, apyrétique avec une sensibilité abdominale à la palpation majorée à l’étage pelvien. L’échographie puis le scanner abdominal mettent en évidence deux masses latéro-utérines, mesurant respectivement 10 cm à droite et 9 cm à gauche (Figure 1). A l’étage sus mésocolique, la vésicule biliaire est multilithiasique à paroi épaissie, circonférentielle, suggérant une cholécystite chronique lithiasique (Figure 2). Le reste du bilan n'a pas montré d'autres lésions. L'exploration chirurgicale révèle un épaississement vésiculaire d'allure plutôt tumorale envahissant le duodénum avec adénopathies hilaires non extirpables. La biopsie extemporanée a révélé un adénocarcinome vésiculaire. Les ovaires sont tissulaires à surface irrégulière nodulaire suggérant des métastases ovariennes. Des biopsies confirment leur nature métastatique dont l'origine est biliaire. La patiente est confiée à l'oncologie pour chimiothérapie palliative. La patiente est décédée 5 mois après.

### Cas 2

**Figure 3:**
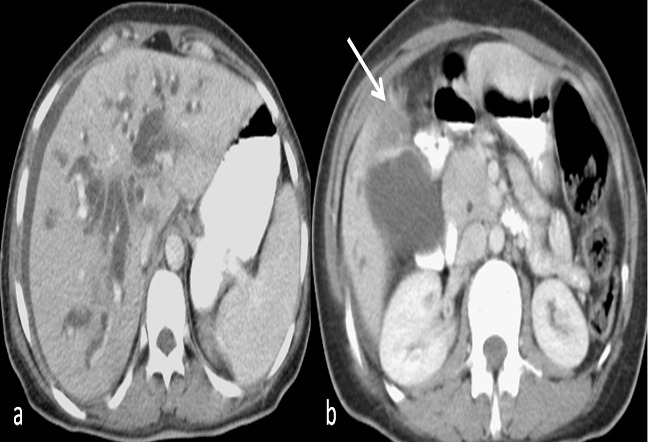
Images scannographiques qui montrent une dilatation des VBIH (a) en amont d'un processus tissulaire vésiculaire d'allure tumorale (b) (flèche).

Patiente âgée de 40 ans, sans antécédents pathologiques particuliers, qui présente des douleurs de l'hypochondre droit avec vomissements et ictère d'allure cholestatique évoluant depuis plus d'un mois. L'examen clinique trouve une patiente en bon état général avec un ictère cutanéo-muqueux. L'examen abdominal révèle une sensibilité de l'hypochondre droit. Le bilan biologique révèle une cholestase et une cytolyse biologique. L'ionogramme et la numération de la formule sanguine sont normaux. L’échographie et le scanner abdominal révèlent la présence d'une masse tumorale de la vésicule biliaire infiltrant le parenchyme hépatique avoisinant et la convergence biliaire avec dilatation des voies biliaires d'amont (Figure 3). La patiente est admise au bloc opératoire pour dérivation biliaire chirurgicale. En per-opératoire, la tumeur envahissait le lit vésiculaire, la voie biliaire principale, le hile hépatique et le colon transverse. Il n'existait pas de métastases hépatiques ni de carcinose péritonéale. Une biopsie de la masse tumorale est revenue en faveur d'un carcinome bien différencié de la vésicule biliaire. La patiente a bénéficié d'une chimiothérapie post-opératoire (Xéloda^®^) en prise continue de 500 mg/j.

**Figure 4:**
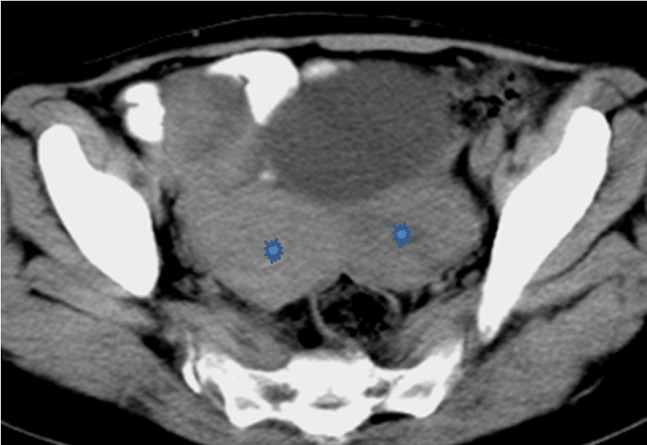
Image scannographique injectée en coupe axiale montrant les métastases ovariennes (étoiles).

Le scanner abdominal de contrôle à 3 mois a objectivé deux masses tissulaires hétérogènes latéro-utérines bilatérales (Figure 4) suggérant fortement le diagnostic de métastases ovariennes. La patiente est décédée 1 mois après.

## Discussion

Les métastases ovariennes ou tumeurs de Krukenberg sont fréquentes avec une incidence qui avoisine 15% [1]. Les sites tumoraux primitifs sont par ordre de fréquence d'origine gastrique (70%), colique (15%) et biliopancréatique (5%) [2, 3]. Les métastases ovariennes d'origine biliaire sont rarement rapportées dans la littérature: Kumar Y. et al a répertorié 19 cas [4] dans la littérature anglophone et Khunamornpong et al a recensé 16 cas en 14 ans [5]. Une localisation, à la fois méningée et ovarienne d'un cancer vésiculaire a été rapportée [6]. Nous rapportons deux cas supplémentaires. La découverte de ces métastases ovariennes se fait de manière concomitante avec le diagnostic de la tumeur primitive ce qui suggère d'emblée leur nature métastatique (cas 1) [7, 8]. Parfois, ces métastases surviennent à distance du diagnostic initial (cas 2) ou encore le précède [5]. En Effet, Maaouni et al a rapporté l'observation d'une métastase ovarienne ayant précédé l'apparition du cancer biliaire [9]. Selon Petru et al, l'atteinte ovarienne peut précéder la détection de la tumeur primitive jusqu'a dans 38% des cas [10] toutes localisations confondues. Et dans ces deux dernières situations, se pose le problème de diagnostic différentiel avec une tumeur ovarienne primitive.

Sur le plan physiopathologique, plusieurs hypothèses ont essayé d'expliquer cette extension parmi lesquelles nous retenons la voie lymphatique rétrograde, le chimiotactisme l'immunotactisme, et hormonotactisme [9, 11].

Cliniquement, les patientes peuvent se présenter à un stade avancé de la maladie avec ictère cholestatique par envahissement locorégional et altération de l’état général. En cas de maladie biliaire locale, les manifestations biliaires peuvent être masquées par les symptômes en rapport plutôt avec la maladie ovarienne (douleurs pelviennes vagues, palpation de masse, ascite).

En imagerie, les métastases ovariennes se présentent sous forme de masses majoritairement tissulaires, siège de quelques les images kystiques intramurales bien limitées [12]. Certains auteurs ont relevé des éléments suggérant la nature métastatique des masses ovariennes: (I) le caractère solide ou mixte de la masse [13], (II) la bilatéralité [13], (III) limites nettes et régulières [14], (IV) extension extraovarienne [7] et (V) une taille inférieure à 10 cm [15]. Plus encore, Choi et al s'est intéressé à l'aspect en imagerie du krukenberg d'origine gastrique versus colique et a conclu que la métastase ovarienne gastrique présente une composante solide majoritaire, se rehausse plus après contraste et est de taille plus réduite [14].

En histologie, l'immunohistochimie montre une positivité au CK 7. La biologie moléculaire est d'un apport fort intéressant et permet de différencier une origine primitive ou secondaire de la masse ovarienne en cas de doute diagnostic [16] et ce par la technique de recherche de perte chromosomique à l'aide de marqueurs microsatellites.

## Conclusion

L'origine biliaire des métastases ovariennes doit être dorénavant incluse dans la liste des tumeurs susceptibles de métastaser dans les ovaires surtout dans les pays qui ont une grande fréquence de tumeurs biliaires.
